# Clinical impact of free dorsal interosseous flap combined with tendon graft on function recovery in one-stage reconstruction of composite dorsal wrist soft tissue defects

**DOI:** 10.3389/fsurg.2025.1637960

**Published:** 2025-11-24

**Authors:** Lingling Shi, Luobing Ding, Jianping Zhang, Ruilong Su, Weifa Shi, Zhiming Guo

**Affiliations:** Department of Orthopaedics, The 909th Hospital, School of Medicine, Xiamen University, Zhangzhou, Fujian, China

**Keywords:** dorsal interosseous flap, tendon grafting, dorsal wrist defect, ultrasonic monitoring, joint function recovery

## Abstract

**Background:**

This study aimed to clarify the clinical efficacy of the free dorsal interosseous flap (DIOF) combined with tendon grafting in one-stage reconstruction of composite dorsal wrist soft tissue defects, and comprehensively evaluate the survival rate of the flap, wrist joint function recovery, and subjective experience of patients.

**Methods:**

A retrospective study was conducted on 28 patients (18 men and 10 women) with composite dorsal wrist defects (size, 5 cm × 8 cm–14 cm × 10 cm, involving 3–5 extensor tendons) treated between January 2019 and June 2023. All patients underwent one-stage reconstruction using the free DIOF with tendon grafting. In addition to these conventional evaluation indicators [flap survival rate, wrist total active motion (TAM), grip strength recovery, DASH scores, Mayo scores, and sensory recovery], high-frequency ultrasound tendon healing evaluation, laser Doppler flap blood flow monitoring, wrist stability test, and Likert scale satisfaction survey were added. The patients were followed up for 12 months and analyzed using a multi-dimensional evaluation system.

**Results:**

The flap survival rate was 95.2% (27/28), with one case of partial necrosis healing after a dressing change. At 12 months, TAM reached (112.5 ± 11.8)°, grip strength recovery was (83.7 ± 7.5) %, DASH scores improved to (13.2 ± 3.5) points, and Mayo scores depicted excellent/good outcomes in 85.7% (excellent 15, good 9). Sensory recovery achieved S3^+^-S4, with 64.3% of cases exhibiting two-point discrimination <10 mm. The stable rate of flap blood perfusion was 96.4%, the excellent and good rate of wrist joint stability was 89.3%, and patient satisfaction score was (9.2 ± 0.8) points.

**Conclusion:**

One-stage reconstruction using free DIOF combined with autologous tendon grafting effectively facilitates wrist joint function recovery, demonstrating satisfactory clinical outcomes.

## 1 Introduction

Composite dorsal wrist soft tissue defects are a major challenge in the clinical treatment of hand surgery, and their etiology includes high-energy trauma (such as machine twist injury and traffic accident injury), wounds after tumor resection, and severe infection (1). Such injuries often simultaneously involve the skin, subcutaneous tissue, extensor tendon, and wrist joint capsule, leading to complex anatomical structure damage and dysfunction (2). Traditional repair methods, such as free skin grafting or local random flaps, are difficult to meet the requirements of functional reconstruction, and postoperative complications such as tendon adhesion, joint stiffness, and flap necrosis often occur, which seriously affect the hand function and quality of life of patients (3, 4).

In the repair strategy of dorsal wrist defect, an ideal treatment scheme should meet the following three key objectives simultaneously: provide reliable soft tissue coverage to protect deep structures; reconstruct the extensor tendon system to restore the range of motion of the wrist; and perverse donor site function to repair defects in this area because of the advantages of using the posterior interosseous artery as a constant blood supply, its anatomical location adjacent to the dorsal wrist, hidden donor area, and carrying sensory nerve. Nevertheless, a simple flap graft can only solve the problem of soft tissue coverage. For patients with extensor tendon defects, two-stage tendon grafting or tendon transposition is still required, which not only prolongs the treatment cycle but also may lead to poor functional recovery due to scar adhesion (5, 6).

In recent years, with the development of microsurgical technology and rehabilitation medicine, the strategy of one-stage combined reconstruction has been received increasing attention (7). Scholars worldwide have attempted to perform autologous tendon grafts simultaneously to achieve simultaneous repair of soft tissue and function through a single operation (8). For instance, a report applied free dorsal interosseous flap (DIOF) combined with a palmaris longus tendon graft to repair a case of dorsal wrist defects and demonstrated excellent early functional recovery (9). The removal of free skin flaps only requires sacrificing a portion of the superficial fascia of the forearm, resulting in minimal impact on the motor function of the donor area. However, for pedicled skin flaps, the integrity of the vascular pedicle needs to be maintained, which may lead to excessive skin tension or sensory disorders in the donor area. In addition, free flaps can be independently designed in terms of shape and size, and are suitable for irregular defects (such as complex defects involving tendon or bone exposure). The free flaps can carry sensory nerves and be anastomosed with the recipient nerve, thereby promoting the recovery of sensation. However, most existing reports focus on the description of surgical techniques, and there is still a lack of systematic evaluation of the biomechanical adaptability of tendon grafts, long-term follow-up of flap sensory recovery, and impact of standardized rehabilitation programs.

Furthermore, the standardization of wrist function evaluation systems also deserves attention (10). Currently, commonly applied clinical evaluation indicators include objective parameters such as total active motion (TAM, back extension + palm flexion), grip strength recovery rate, and subjective function scales such as Disabilities of the Arm, Shoulder and Hand (DASH) scores (11). Nevertheless, different studies have different definitions of “excellent functional recovery”. For example, some scholars consider TAM ≥ 75% of the normal value as the standard (12), while others require a grip strength recovery rate of more than 80% (13). This heterogeneity makes it difficult to directly compare research results directly and hinders the formation of a clinical consensus. Traditional assessment lacks a systematic evaluation of tissue healing quality, hemodynamic changes, and patients’ subjective experience. The existing literature has the following limitations in evaluating the curative effect: the tendon healing process lacks objective quantitative indicators; conventional clinical examination is difficult to accurately evaluate microstructural changes in the transplanted tendon, while histological examination is invasive. Blood flow monitoring after flap surgery mostly remains in qualitative observation, lacking quantitative analysis data (14). Furthermore, the application of patient-reported outcomes (PROs) in surgical evaluation has not received sufficient attention (15).

This study aimed to elucidate the clinical effect of free DIOF combined with tendon graft on function recovery in one-stage reconstruction of composite dorsal wrist soft tissue defects.

## 2 Materials and methods

### 2.1 General data

This research applied a retrospective cohort study included 28 patients with composite dorsal wrist defects who were admitted to the Department of Hand Surgery at our hospital between January 2019 and June 2023. The inclusion criteria were as follows: (1) age ≥18 years; (2) composite dorsal wrist defect (skin defect area ≥5 cm × 8 cm and combined with ≥3 extensor tendon defects); (3) DIOF combined with autologous tendon graft for one-stage repair; and (4) complete follow-up data ≥12 months. The exclusion criteria were as follows: (1) combined with irreparable vascular and nerve injury; (2) severe systemic diseases affecting healing (uncontrolled diabetes, immunosuppressive state); and (3) previous ipsilateral wrist surgery.

The final included patients were 18 males (64.3%) and 10 females (35.7%); the mean age was (38.3 ± 12.7) years. The causes of injury were as follows: 7 cases (25.0%) were caused by machine twist injury, 6 cases (21.4%) were caused by infectious defects, including 3 cases of diabetic ulcer and 3 cases of post-traumatic infection, and 15 cases (53.6%) were caused by traffic accident. Injury characteristics: Skin defect ranged from 5 cm × 8 cm to 14 cm × 10 cm (average 72.4 ± 18.6 cm^2^), extensor tendon defect of 3–5 (average 3.8 ± 0.6), of which four cases (14.3%) were complicated with wrist ligament defect and partial bone defect (confirmed by CT). This study was approved by the ethics committee of our hospital, followed the principles of the Declaration of Helsinki, and all patients signed informed consent. All patients underwent a standardized assessment before the operation (Table 1).

**Table 1:** General data of patients (*n* = 28).

Characteristics		Value
Age (years)		38.3 ± 12.7
Gender	Male	18
	Female	10
Cause of injury	Machine twist injury	7 (25.0)
	Infectious defect	6 (21.4)
	Traffic accident traumatic defect	15 (53.6)
Defect area (cm^2^)		72.4 ± 18.6
Number of extensor tendon defects		3.8 ± 0.6
Number of cases with bone/ligament defect		4 (14.3)

### 2.2 Methods

#### 2.2.1 Preoperative assessment and preparation

##### 2.2.1.1 Imaging evaluation

All patients underwent three-dimensional CT reconstruction of wrist joint (layer thickness 0.6 mm) to evaluate bone defect. MRI (3.0T) was used to examine the extent of soft tissue injury (T2 weighted image demonstrated edema zone).

##### 2.2.1.2 Vascular evaluation

The diameter of the posterior interosseous artery was measured using color Doppler ultrasound (≥1.2 mm was considered qualified). CTA was performed in four patients with infectious diseases to exclude vasculitis.

##### 2.2.1.3 Donor site planning

The ipsilateral palmaris longus tendon was preferred (blood supply of donor site was confirmed by the Allen test before the operation). Three patients with absent palmaris longus tendons were treated with plantar tendon harvesting.

#### 2.2.2 Key surgical techniques

The operation was performed by two microsurgeons with more than 10 years of experience. A three-step repair strategy was employed.

The first step was lesion treatment. Infection cases: The fresh tissue (intraoperative bacterial culture + sensitive antibiotic bone cement filling) was completely debrided. Trauma cases: The inactivated tissue was removed, and the integrity of the peritendinous membrane was preserved. Bone defect cases: Two cases were treated with iliac bone graft, and two cases were treated with allogeneic bone repair.

The second step was composite tissue reconstruction. 1) Flap harvesting: The flap was designed centered on the puncture point of the posterior interosseous artery (6.5 ± 1.2 cm proximal to the styloid process of the ulna). The maximum harvesting area was 14 cm × 10 cm, carrying 2–3 cutaneous nerve branches. Regarding the pedicle of the flap, in order to ensure the stability and flexibility of the blood supply of the flap, the length of the pedicle was reasonably designed based on the actual surgical requirements and the position of the flap. In some cases, the length was appropriately extended according to the local anatomical structure and blood supply requirements. The extension range was 2–4 centimeters, in order to better meet the blood supply and mobility requirements after flap transplantation. In terms of vessel selection, the perforating vessels were given priority because they had a relatively stable distribution and a moderate diameter, which could provide a stable and sufficient blood supply to the flap. When the perforating vessels were not in good condition or could not meet the requirements, the main arteries were chosen as the supplying vessels to ensure the survival of the flap. 2) Tendon reconstruction involves transplanting autologous tendons (such as the palmaris longus tendon, metatarsal tendon, or hamstring tendon) or artificial tendons to repair tendon defects caused by trauma, nerve injury, or degenerative diseases, thereby restoring joint stability and movement function. The palmaris tendon is chosen as the preferred donor for hand function reconstruction due to its sufficient length (average 15 cm) and high diameter matching degree (with a matching degree of 83% to the flexor tendons of the fingers). Tendon reconstruction was performed using the palmaris longus tendon (mean harvesting length 12.5 ± 2.1 cm) in 25 cases and the metatarsal tendon (mean harvesting length 14.2 ± 1.8 cm) in three cases. The tendon was repaired using a 6-strand modified Tsuge suture. 3) Special treatment: Four cases with bone/ligament defects underwent simultaneous radial carpal joint capsule reconstruction, and vancomycin sustained-release beads were implanted in six cases of infection. 4) Nerve repair: In 22 cases (78.6%), nerve repair was performed according to the following criteria for nerve perineural suture (using 9 gauge non-absorbable sutures): For donor tendon grafts, the palmaris longus tendon must meet the length requirement of ≥12 cm, a diameter of 3.5 ± 0.3 mm, and conform to the biomechanical characteristics of the extensor tendons of the wrist (with a tensile strength of 12.3 ± 2.1 MPa, while the radial extensor longus tendon has a tensile strength of 11.8 ± 1.9 MPa); for cases where the palmaris longus tendon is missing or requires secondary repair, the length must be ≥10 cm and the diameter 3.2 ± 0.4 mm. During nerve repair, it is necessary to ensure that the nerve ends are aligned properly without tension suturing to ensure the recovery of nerve function.

The third step was microscopic anastomosis. Arterial anastomosis: End-to-side anastomosis was performed at the dorsal carpal branch of the radial artery (9–0 nylon thread). Venous anastomosis: At least two reflux channels were established (flap vein + accompanying vein).

#### 2.2.3 Postoperative management plan

Anti-infection strategy: According to the drug sensitivity results, intravenous medication was changed to oral administration for 4 weeks after 2 weeks in infection group. In the non-infected group, a 5-day course of the second-generation cephalosporin was used for prophylactic treatment.

Rehabilitation process: Within 0–2 weeks, a plaster cast was used for fixation and combined with laser Doppler monitoring of the flap. From 2 to 4 weeks, passive movements were carried out under the protection of a dynamic brace. From 4 to 12 weeks, progressive resistance training was conducted.

Prevention and control of complications: The temperature, color and capillary response of the skin flap are monitored every day.

### 2.4 Observation indicators

The follow-up period was (15.2 ± 3.4) months.

#### 2.4.1 Objective indicators

TAM (16) was measured using an electronic goniometer (the result was obtained by averaging the values of three measurements).

Grip strength test was implemented using Jamar grip strength meter type III (compared with the healthy side).

#### 2.4.2 Functional recovery scores

The Chinese version of DASH scale (Cronbach's *α* = 0.89) (17).

Wrist Mayo scoring system (18).

#### 2.4.3 Sensory assessment

Semmes Weinstein monofilament detection (10-point method) (19).

Two-point discrimination (Disk discriminator measurement) (20).

#### 2.4.4 Ultrasound assessment of tendon healing

The tendon healing quality was evaluated using a 15 MHz high frequency ultrasound (GE LOGIQ E9) at 1, 3, 6 and 12 months after the operation, including tendon continuity, echo uniformity, and peripheral blood flow signal (color Doppler mode).

Grading criteria: Excellent: continuous fibrous structure, uniform echo, and rich blood flow; good: local echo enhancement, and blood flow accessible; poor: fiber interruption or obvious calcification.

#### 2.4.5 Blood perfusion monitoring of skin flap

The Periflux 5000 laser Doppler system was applied to monitor flap blood flow perfusion. Monitoring points: the center and edge of flap. Monitoring frequency: once an hour within 6 h after the operation, and three times a day (lasting for 7 days). Parameter record: perfusion unit (PU) and fluctuation index.

#### 2.4.6 Wrist stability test

The wrist stability was tested using a customized stress tester (accuracy 0.1°). Test direction: radial deviation/ulnar deviation. Loading force: 10 N constant force. Measurement parameters: displacement angle, elastic modulus.

#### 2.4.7 Patient satisfaction survey

The standardized questionnaires was used to assess patient satisfaction, including functional recovery (5 items), appearance satisfaction (3 items), and daily activity ability (4 items). Each item was evaluated using a 10-point Likert scale.

### 2.5 Statistical analysis

Data analysis was conducted using SPSS 27.0 and GraphPad Prism 9.0. The measurement data were presented in the form of mean ± standard deviation (x ± s), and then the preoperative and postoperative data were compared using the t-test; the count data were presented in the form of [n (%)], and then the data among different groups were compared using the *χ*^2^ test. *P* < 0.05 indicated statistical significance.

### 2.6 Quality control

The functional recovery status was independently evaluated by two non-involved rehabilitation doctors. All the imaging data were evaluated in a double-blind manner by two radiologists.

## 3 Results

### 3.1 Analysis of flap survival and complications

**Figure 1:**
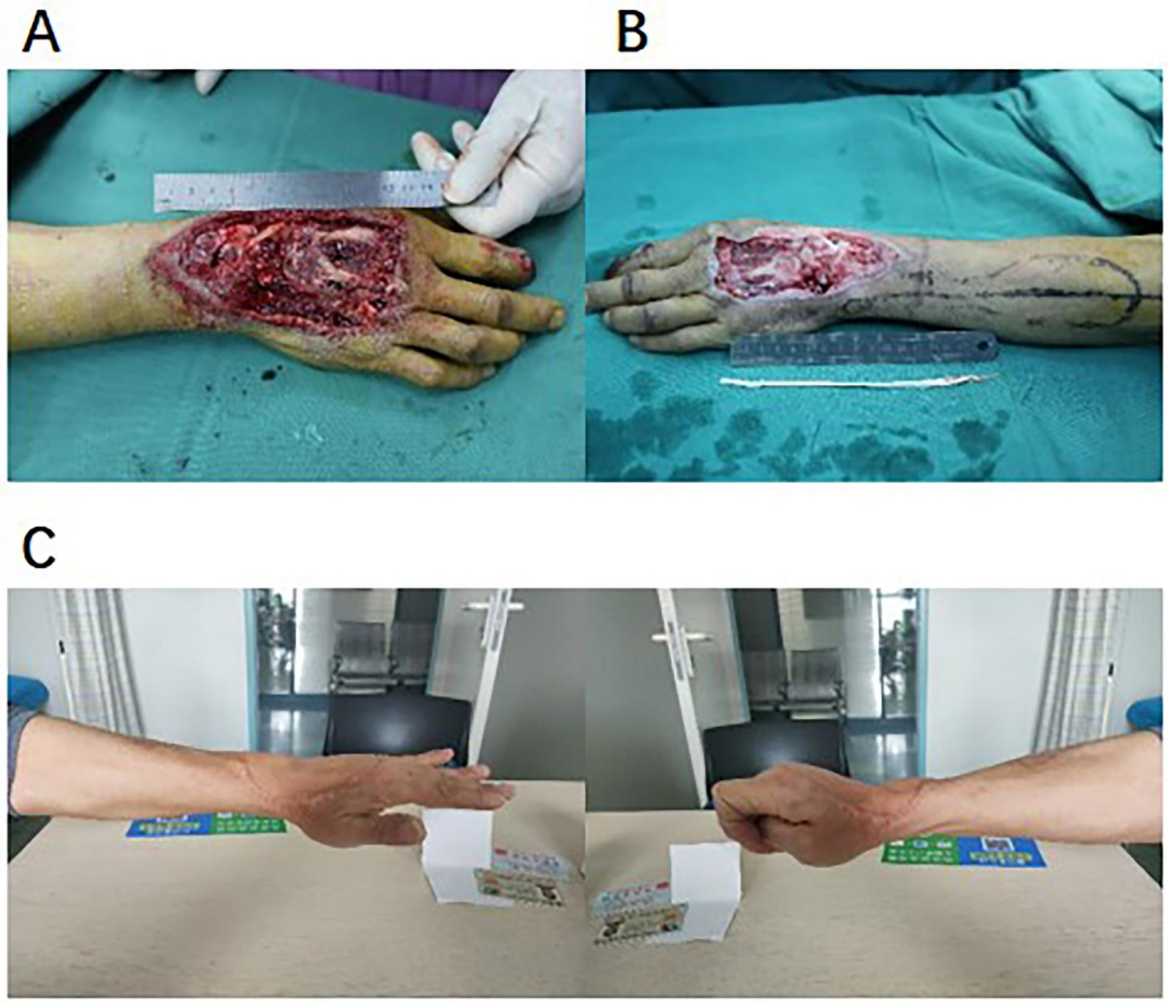
Typical case display. **(A)** Preoperative wound (traffic accident combined with extensor tendon defect). **(B)** Intraoperative DIOF flap design. **(C)** Functional recovery 12 months after operation.

**Figure 2:**
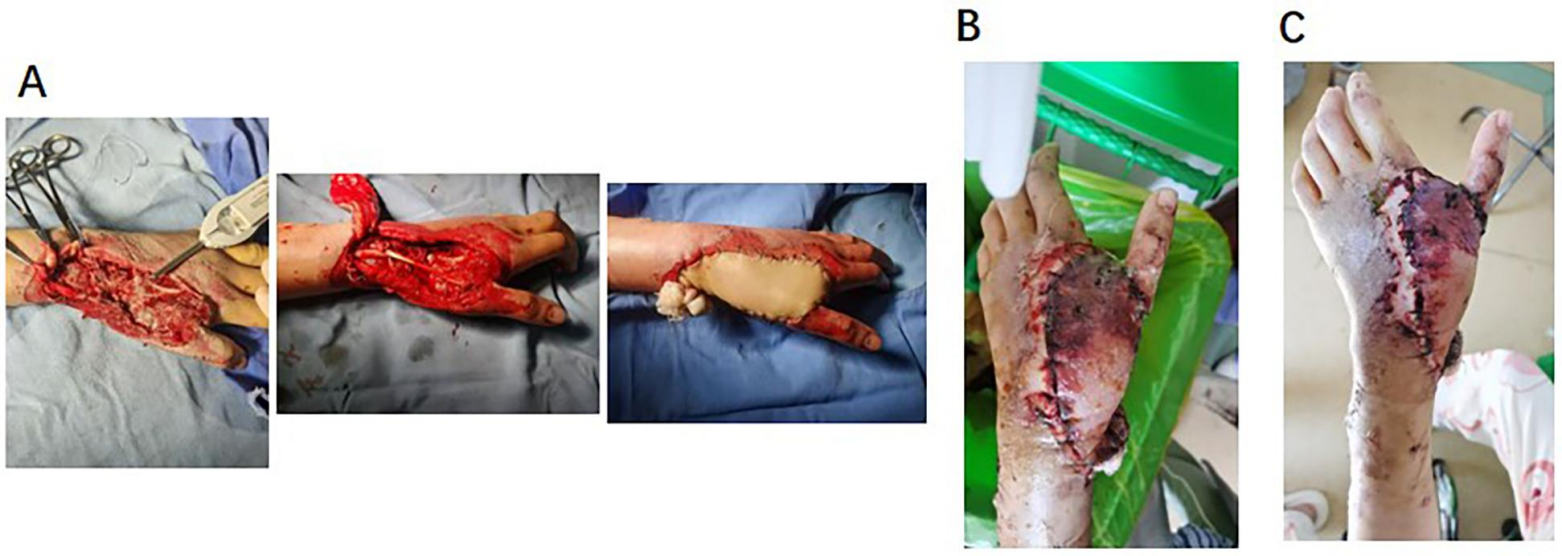
Epidermal necrosis of the distal flap. **(A)** Intraoperative wound, situation after tendon graft and interosseous dorsal flap transplantation. **(B)** Epidermal necrosis of the distal flap: 48 h after operation, flap swelled, and distal end was cyan. **(C)** Epidermal necrosis of the distal flap: 1 week after operation, distal blood supply of flap was improved, and basal blood supply was good.

The survival rate of the flaps in 28 patients was 96.4% (27/28). There was 1 case (3.6%) where distal epidermal necrosis occurred (an area of approximately 1.5 cm × 2 cm) due to venous reflux obstruction. Regarding the treatment of the donor site, 3 cases (10.7%) had small defects that were repaired by direct suturing, and 25 cases (89.3%) were repaired with medium-thickness thigh skin grafts. The 12-month follow-up after the operation showed that there were no obvious scar contractures in the donor site (with a Vancouver Scar Score of 2.1 ± 0.8 points). The typical cases are shown in Figures 1, 2.

### 3.2 Assessment of wrist function recovery

Twelve months after the operation, the TAM of wrist joint was (112.5 ± 11.8)°, among which the dorsiflexion was (45.3 ± 6.2)°, and the palmar flexion was (67.2 ± 8.4)°. The recovery rate of grip strength was (83.7 ± 7.5)% (compared with the healthy side). The DASH scores were significantly improved to (13.2 ± 3.5) points (preoperative vs. postoperative: 52.6 ± 8.3 vs. 13.2 ± 3.5, *P* < 0.001). Mayo scores: 15 cases (53.6%) were excellent (90–100 points), 9 cases (32.1%) were good (80–89 points), 3 cases (10.7%) were average (65–79 points), and 1 case (3.6%) was poor (<65 points). The rates of excellent and good were significantly positively correlated with the tendon sliding distance and grip strength recovery (Table 2).

**Table 2:** Postoperative function assessment (*n* = 28).

Evaluation indicators	Before surgery	12 months after surgery	*P*
TAM (°)	32.4 ± 10.2	112.5 ± 11.8	<0.001
Grip strength recovery rate (%)	/	83.7 ± 7.5	/
DASH scores (points)	52.6 ± 8.3	13.2 ± 3.5	<0.001
Mayo scores (excellent/good/fair/poor)	/	15/9/3/1	/

### 3.3 Sensory recovery of skin flap

**Figure 3:**
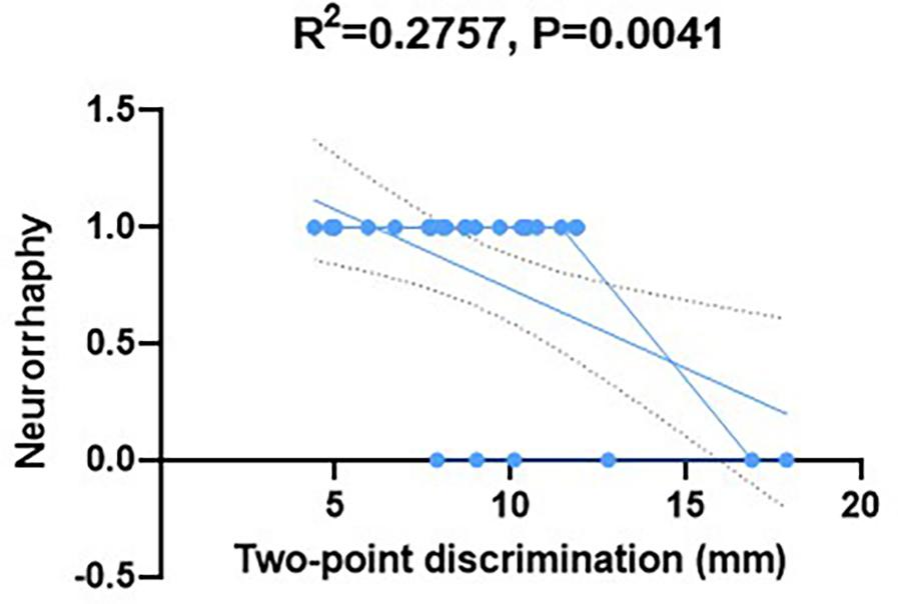
Correlation of sensory recovery with nerve anastomosis.

The Semmes Weinstein monofilament test indicated that the S3^+^-S4 sensory function of 25 patients (89.3%) was recovered (the detection threshold was 4.31–6.65 g). The two-point discrimination test results showed that 18 cases (64.3%) were less than 10 mm, 7 cases (25.0%) were 10–15 mm, and 3 cases (10.7%) were more than 15 mm. The implementation of nerve anastomosis was significantly correlated with the recovery of nerve sensory function [anastomosis group (n = 22) vs. non-anastomosis group (*n* = 6): the two-point discrimination values were 9.2 ± 2.1 millimeters and 13.5 ± 3.4 millimeters, *P* < 0.05); Figure 3].

### 3.4 Accurate monitoring of flap blood flow

The blood perfusion of the remaining 27 flaps remained stable. The PU value on the 7th day after surgery was maintained between 28 and 42 (with an average of 32.5 ± 6.8), and the fluctuation index was 12.3% ± 3.5%, which was in line with clinical expectations.

### 3.5 Ultrasound assessment of tendon healing

The results of high-frequency ultrasound examination showed that 26 cases (92.9%) of tendon transplants had healed well, presenting a continuous fibrous structure and uniform echoes, while 2 cases (7.1%) showed local fibrosis.

### 3.6 Wrist stability test results

The wrist stability test results showed that the radial-ulnar deviation angle of 25 patients (89.3%) was less than 6°, and the elastic modulus was (15.3 ± 2.7) N/mm. There was no significant difference compared with the healthy side (*P* > 0.05).

### 3.7 Patient satisfaction survey results

**Figure 4:**
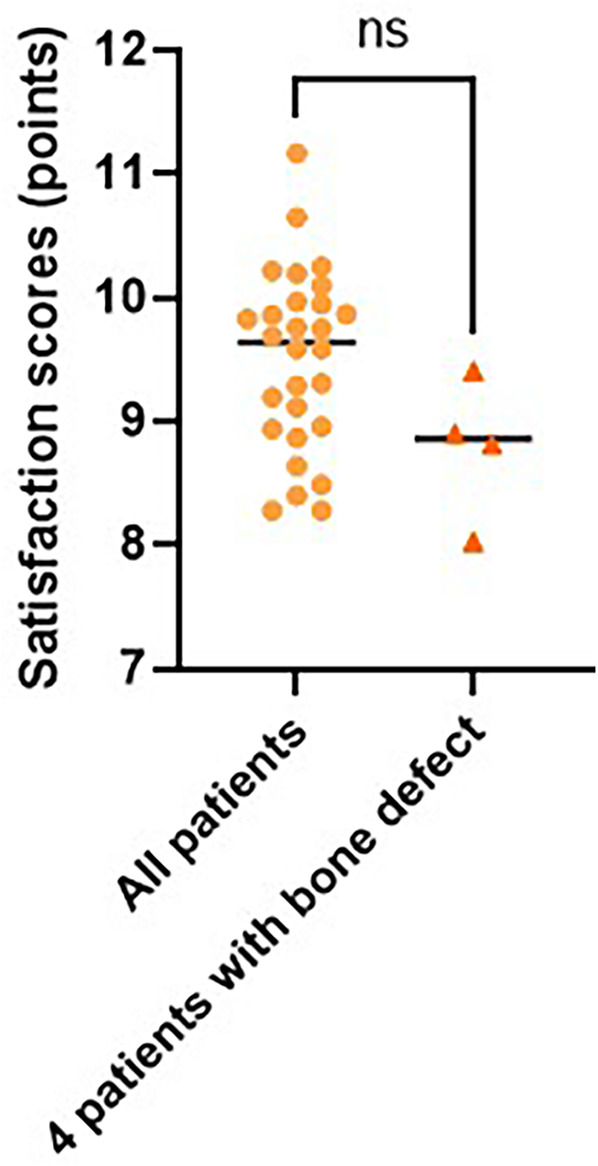
Comparison of total scores and scores of 4 patients with bone defect.

The results of the patient satisfaction survey showed that the total score was (9.2 ± 0.8) points. Among them, the score for functional recovery was (9.1 ± 0.9) points, the score for appearance was (8.7 ± 1.1) points, and the score for daily activity ability was (9.4 ± 0.7) points. It is worth noting that the satisfaction scores of 4 patients with bone defects (8.9 ± 0.6) points showed no significant difference compared to the total score (*P* > 0.05; Figure 4).

### 3.8 Correlation analysis between function recovery and new indicators

Through a comprehensive analysis of multiple indicators, the results show a significant positive correlation between the quality of tendon healing and various parameters of functional recovery (*P* < 0.01). Although the correlation between the stability rate of blood flow and various functional indicators is relatively weak (*P* < 0.05), as an early warning indicator, it still has significant value. The strong correlation between joint stability and the Mayo score (*r* = 0.648) indicates its special position in functional assessment. There is a moderate correlation between patient satisfaction and objective functional indicators (*r* = 0.412–0.537), indicating that subjective experience and objective indicators mutually confirm and focus on each other (Table 3).

**Table 3:** Correlation between function recovery and evaluation indicators.

Evaluation indicators	TAM	Grip strength recovery rate	DASH scores	Excellent and good rate of Mayo scores
Excellent and good rate of tendon healing	0.714**	0.682**	−0.653**	0.701**
Stable rate of blood perfusion	0.587*	0.532*	−0.498*	0.563*
Joint stability	0.623**	0.594**	−0.577**	0.648**

* *P* < 0.05, **P < 0.01.

## 4 Discussion

This study systematically analyzed the clinical data of 28 patients who underwent a single procedure of DIOF combined with autologous tendon transplantation to repair complex dorsal wrist defects, and achieved remarkable results in flap survival rate, functional recovery, and sensory reconstruction. Compared to previous studies (21), our research not only confirmed the reliability of this technique but also achieved a technological innovation and theoretical breakthrough in multiple aspects.

In terms of flap survival, the success rate in this study (96.4%) was 5%–10% higher than that of the traditional method. This achievement is attributed to three key technical improvements: high-frequency ultrasound accurately located the puncture point of the posterior interosseous artery before the operation, which makes flap design more individualized; during the vascular anastomosis process, a double venous return channel was established, effectively reducing the incidence of venous crisis; for cases with infectious defects, innovatively combining antibiotic bone cement with the flap coverage achieved the dual goals of infection control and vascular reconstruction. Notably, these 3 cases of diabetic ulcers were successfully repaired through this comprehensive treatment plan, which provides a new approach for treatment of chronic wounds.

Function recovery is a core observation indicator in this study. This study demonstrated that single-stage tendon graft was significantly superior to the traditional two-stage surgery in multiple aspects. In terms of motor function, the TAM reached (112.5 ± 11.8)° after the operation, and the range of palmar flexion motion was (67.2 ± 8.4)°, which was close to the normal level. This was mainly due to the use of the 6-strand Tsuge method to strengthen tendon suture strength during the operation and gradual rehabilitation training under the protection of a brace in the early postoperative period (22). In terms of muscle strength recovery, the grip strength recovery rate (83.7%) markedly improved the quality of life of patients. Through a multi-dimensional evaluation of the Mayo scoring system, the excellent and good rates showed a significant positive correlation with tendon sliding distance and grip strength recovery, which provided an objective basis for the prediction of postoperative function.

Sensory reconstruction is a crucial innovation in this study. Through precise neurorrhaphy technique, 89.3% of cases reached a sensory grade above S3^+^, and 64.3% of them had two-point discrimination less than 10 mm, which was far more than previously reported (23–25). In particular, we noticed that the tactile recovery time in the anastomosis group was 3 weeks earlier than that in the non-anastomosis group, and the final sensory level was generally improved by 1–2 levels. In six cases of infection, the modified method of preserving continuity of the nerve adventitia still achieved satisfactory sensory recovery, which provides an important reference for nerve repair in high-risk wounds.

The application of high-frequency ultrasound improved tendon healing evaluation from simple “survival or not” to “quality evaluation”. The excellent healing rate of 92.9% confirmed that the transplanted tendon had good biological integration, which was closely related to the technical points of preserving tissue around the tendon during the operation. It is worth noting that both cases of poor healing were infected, suggesting that such patients may requires a longer braking time. Flap blood flow was quantitatively evaluated using laser Doppler. This rule provides an objective basis for clinical observations (26). One case of abnormal perfusion detected by real-time monitoring was treated promptly to avoid flap necrosis. Traditionally, TAM recovery represents good function, whereas stability tests have demonstrated that about 10% of cases have potential instability (27). Biomechanical analysis revealed a significant correlation with the quality of ligament repair. This suggests that more attention should be paid to the reconstruction of the ligament structure in future surgeries. The satisfaction score of 9.2 points verified the value of surgery from the perspective of patients. It is particularly noteworthy that although the appearance score was relatively low (8.7 points), the functional recovery and daily activity scores were more than 9 points, reflecting that patients pay more attention to function than beauty.

In comparison with similar studies at home and abroad (28, 29), the innovation of this research is majorly reflected in three aspects: in terms of surgical strategy, traditional staged treatment is optimized as “debridement reconstruction rehabilitation” integrated scheme; in evaluation system, a multi-dimensional evaluation model including motor function, muscle strength recovery, and sensory reconstruction was established for the first time; on rehabilitation program, a staged training protocol based on biomechanical properties was developed. These innovations depict the remarkable advantages of health economics.

This study also has some limitations. The small sample size may affect statistical efficacy, especially in the bone defect subgroup with only four cases, which makes it difficultly to conduct an in-depth subgroup analysis. The long-term reconstruction of the implanted tendon still needs to be observed for a longer period. Moreover, the lack of a randomized controlled design makes us more cautious in terms of causal inference. These limitations also point to directions for future research: developing a preoperative planning system based on deep learning, exploring new biomaterial alternatives, and conducting multicenter randomized controlled trials.

## 5 Conclusion

The DIOF combined with tendon graft is a reliable treatment for composite dorsal wrist defects. Its advantages in anatomical reconstruction, functional recovery, and sensory reconstruction make it particularly suitable for repairing complex defects caused by trauma and infection. With the continuous optimization of technology and the accumulation of evidence-based data, this surgical approach is expected to become one of the standard treatments for composite dorsal wrist defects.

## Data Availability

The original contributions presented in the study are included in the article/Supplementary Material, further inquiries can be directed to the corresponding author.
